# Plasmon-Enhanced Light Absorption in (*p-i-n*) Junction GaAs Nanowire Solar Cells: An FDTD Simulation Method Study

**DOI:** 10.1186/s11671-021-03603-1

**Published:** 2021-09-20

**Authors:** E. A. Dawi, A. A. Karar, E. Mustafa, O. Nur

**Affiliations:** 1 Nonlinear Dynamics Research Centre (NDRC), Ajman University, P.O. Box 346, Ajman, United Arab Emirates; 2 Edith Cowan University, 270 Joondalup Drive, Joondalup, WA 6027 Australia; 3 Department of Science and Technology (ITN), Linköping University, Campus Norrköping, 601 74 Norrköping, Sweden

**Keywords:** GaAs nanowire, Au nanoparticles, Surface plasmon, Optical simulation, Field enhancement, Solar cells, Photoconversion

## Abstract

A finite-difference time-domain method is developed for studying the plasmon enhancement of light absorption from vertically aligned GaAs nanowire arrays decorated with Au nanoparticles. Vertically aligned GaAs nanowires with a length of 1 µm, a diameter of 100 nm and a periodicity of 165–500 nm are functionalized with Au nanoparticles with a diameter between 30 and 60 nm decorated in the sidewall of the nanowires. The results show that the metal nanoparticles can improve the absorption efficiency through their plasmonic resonances, most significantly within the near-bandgap edge of GaAs. By optimizing the nanoparticle parameters, an absorption enhancement of almost 35% at 800 nm wavelength is achieved. The latter increases the chance of generating more electron–hole pairs, which leads to an increase in the overall efficiency of the solar cell. The proposed structure emerges as a promising material combination for high-efficiency solar cells.

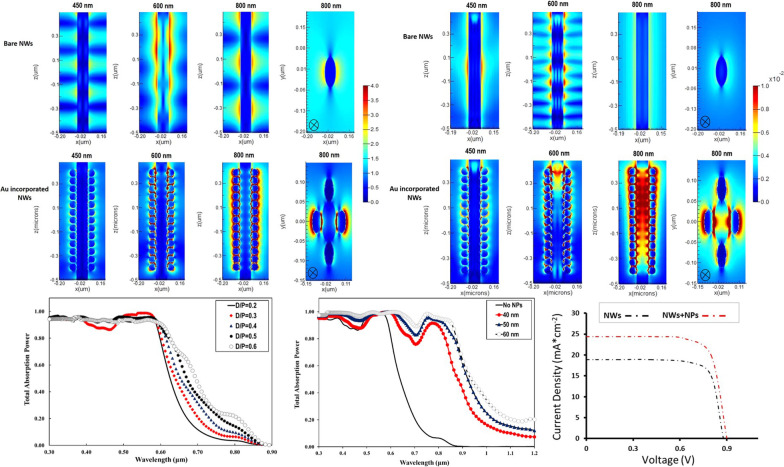

## Introduction

In the quest for renewable energy sources, conventional thin-film photovoltaics (PV) has emerged as promising candidates for commercially viable sources [1, 2]. However, material deficiencies, including dislocations, and poor absorption of thin-film pose major limitations to the performance of such PV cells [3]. To overcome these limitations, significant research and development efforts have been directed toward new emerging PV technologies [4–11]. These technologies have the potential to disrupt and replace the prevailing conventional PV market in the future through the use of advanced light absorption techniques [12–14]. In this context, plasmonic metal nanoparticles (NPs), and their oxides, both in random and periodic distribution, have been found to enhance the generated photocurrent when combined with photodiodes [15, 16], photodetectors [17, 18], solar cell design [10, 11, 19–22], and hybrid organic solar cells [23, 24].

In the search for an innovative approach to reducing the size and cost of solar PV, nanorods and/or nanowires (NWs) have attracted much scientific attention in recent years as exciting new building blocks of solar structures [25, 26]. Their exciting optical and electrical properties, such as high absorption coefficient, direct bandgap, faster charge carrier separation, and higher lateral conductivity than the three-dimensional crystal, have led to improved solar efficiency [27, 28]. Many of III–V semiconductor nanowires hold impressively high carrier mobilities for high-speed devices [29] and flexible electronics [30–32]. In combination with plasmonic NPs, the light trapping ability within these one-dimensional NWs is expected to be improved. In general, miniaturization of the solar cell design is found to shifting both, absorption and fluorescence spectra in nanowires, indicating the generation of multiple localized exciton states [33]. Despite a large number of literature publications, studies on material combinations with plasmonic semiconducting NWs as active systems have rarely been investigated, even less in III–V based semiconducting NW structures. Few scientific experiments have been performed in plasmonic enhanced III–V NWs based solar cells [34–36].

In the current study, the finite-difference time-domain (FDTD) simulation method (Lumerical software package) is used to investigate the effect of plasmons on the optical response of axial p-i-n junction gallium arsenide nanowires (GaAs NWs) based solar cell structures. We optimized the performance of the solar structure by employing different NW *D*/*P* ration decorated with different Au nanoparticles with size between 30 and 60 nm diameters. Our goal is to estimate the electromagnetic fields (EM-fields) that enable strong light coupling using an excitation plasmon-enhanced light-trapping approach. This uses the incorporation of Au metal NPs, which have relatively stable optical properties, to promote light and thus increase cell efficiency. The novelty of this work is one parallel implementation of an effective and practical method which could facilitate the fabrication of high-efficiency GaAs NW solar cells. The advancement of our work lies on the special attention paid to areas where the EM field is highly concentrated at the interface regions of two adjacent NP-NW combinations.

## Materials and Methods

**Fig. 1:**
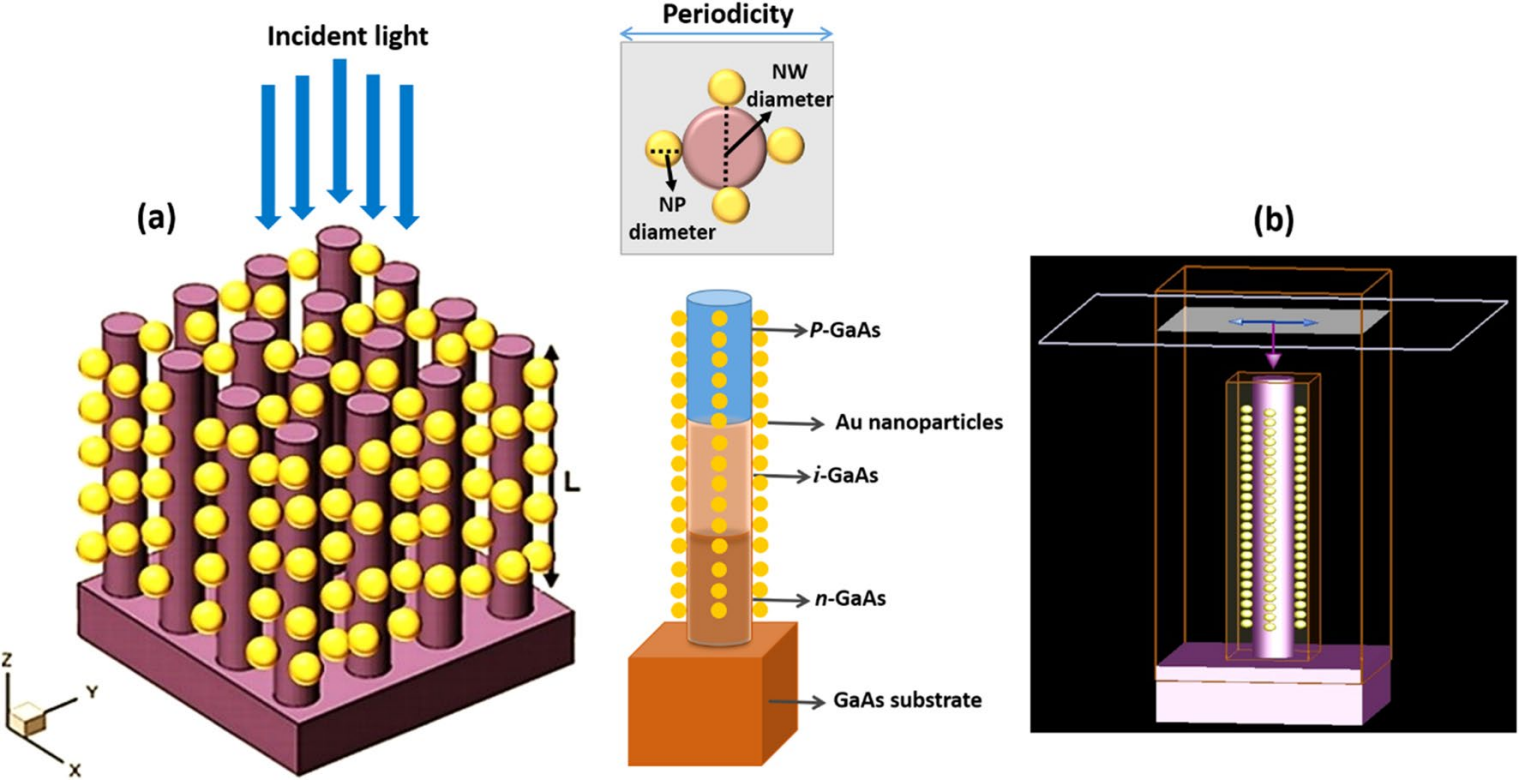
**a** The structure of the plasmonic GaAs nanowire solar cell decorated with Au nanoparticles in 3-D and **b** the simulated unit structure of the plasmonic GaAs nanowire solar cell. The insets represent the top view of a single GaAs nanowire decorated with Au nanoparticles (top) and the *p*-*i*-*n* junction nanostructure (bottom)

Figure 1a, b shows illustrations of our proposed structure of the plasmonic GaAs nanowire solar cell. Each cell contains a periodic NW array, of which a single NW is shown. The structure comprises periodic GaAs nanowires with p-i-n junction with a diameter (*D* = 100 nm) and periodicity (*P* = 100–500 nm), whose sidewall surface is decorated with gold nanoparticles (Au NPs) with diameter between 30 and 60 nm (Fig. 1a). The total length of the nanowires was optimized (*L* = 1 µm) to reduce the dark current, which scales with the NW length. In the current study, GaAs nanowires are simulated in an underlying GaAs substrate. For all simulations performed, Au NPs are incorporated into the NW solar cell structure at the NW sidewall in a uniformly distributed array so that light is coupled into the NWs from all directions, as shown in Fig. 1b. Au NPs with diameters between 30 and 60 nm are incorporated into the NW solar cell structure. The simulations are performed with periodic boundary conditions in the *x*–*y* directions to ensure the periodicity of the entire structure. Moreover, the simulation domain is closed at the top and bottom with an optically suitable transparent layer to allow both reflected and transmitted light to exit the simulation volume. The reflection and transmission monitors are located on the top and bottom of the GaAs NWs, respectively. To ensure coherent results, the amount of power transmitted through the power monitors is normalized to the source power for the entire simulated wavelength range. In addition, the AM1.5G solar illuminator is used to represent the incident light from the top and is set parallel to the GaAs NW axis (in-*z* direction). A plane wave of incident power intensity with wavelengths from 300 to 1000 nm is used, covering the absorption range of the GaAs material. The material critical parameters for the structure simulations such as minimum mobility, SRH lifetime, effective density of states, Auger coefficient, surface recombination velocity and the dispersion properties of GaAs were mostly taken from the literature [37, 38]. The electrical modeling was partially performed using the Sentaurus Electromagnetic Wave Solver (EMW) and *S*-device solver module packages, taking into account the main physical properties of GaAs. The optical generation profiles are integrated into the finite element mesh of the NWs in the electrical tool.

## Results and Discussion

**Fig. 2:**
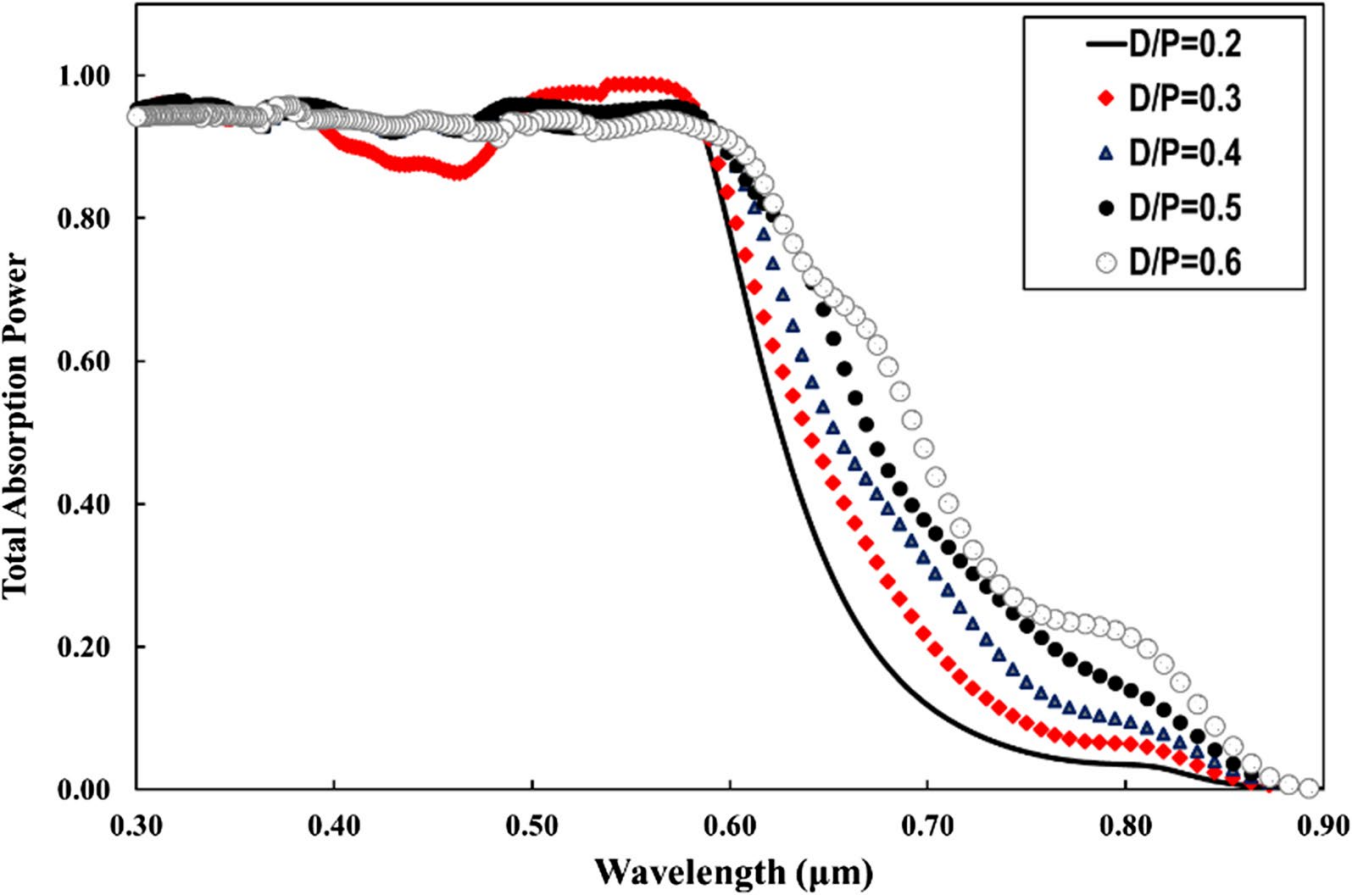
The total absorption performance of GaAs NW with different *D*/*P* ratios without incorporation of Au metal nanoparticles

**Fig. 3:**
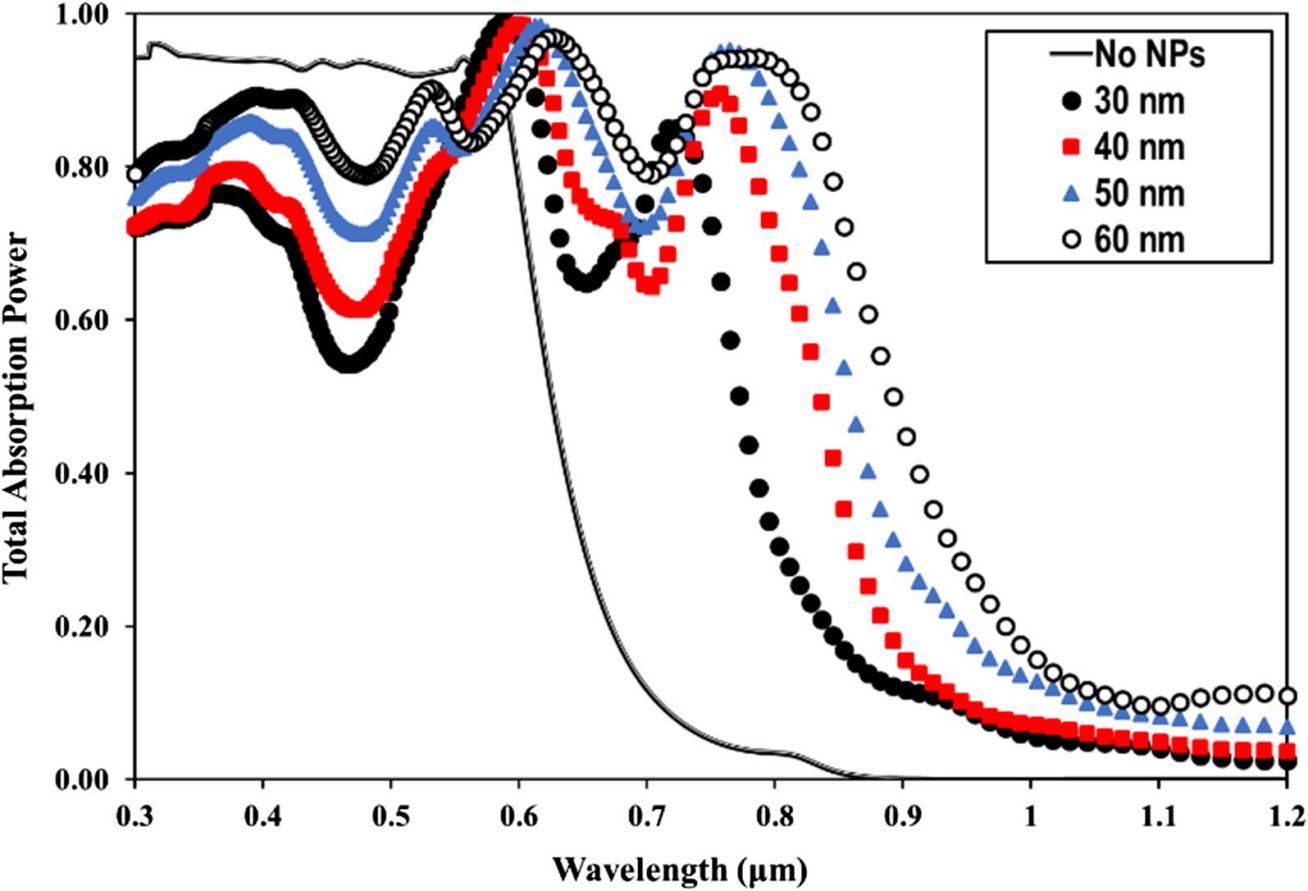
The total absorption efficiency of GaAs NW with *D*/*P* ratios of 0.2 (**a**) incorporated with different Au NP sizes from 30 to 60 nm in diameter compared to bare NW

**Fig. 4:**
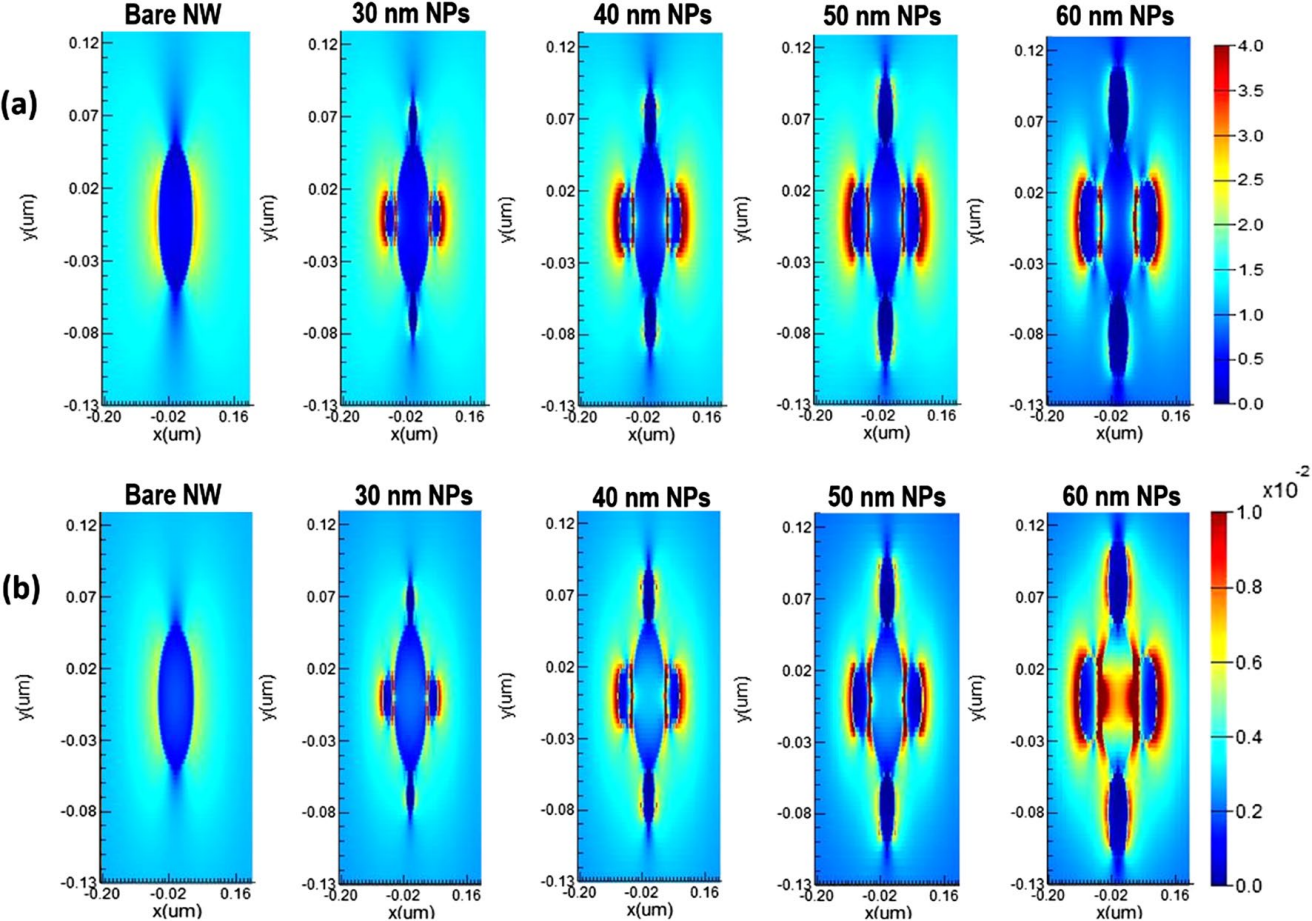
Top view of the 2D light distribution; **a** the calculated absorbed power; **b** the GaAs nanowire after incorporation of the Au NPs of 30, 40, 50 and 60 nm diameter calculated by FDTD at the light wavelength of 800 nm compared to the bare GaAs NW

**Fig. 5:**
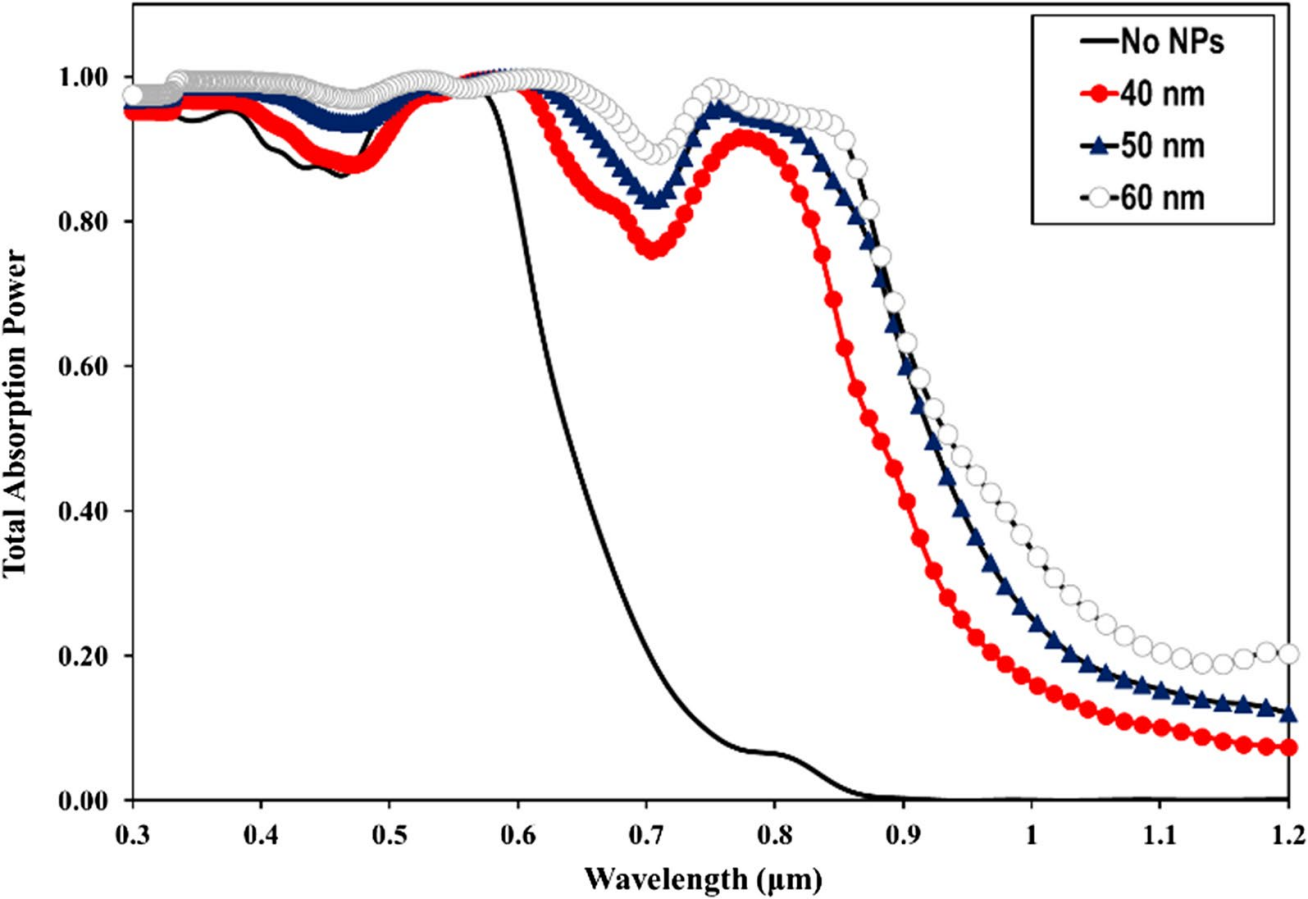
The total absorption efficiency of GaAs NW with *D*/*P* ratios of 0.3 incorporated with different Au NP sizes from 40 to 60 nm in diameter compared to the bare NW

**Fig. 6:**
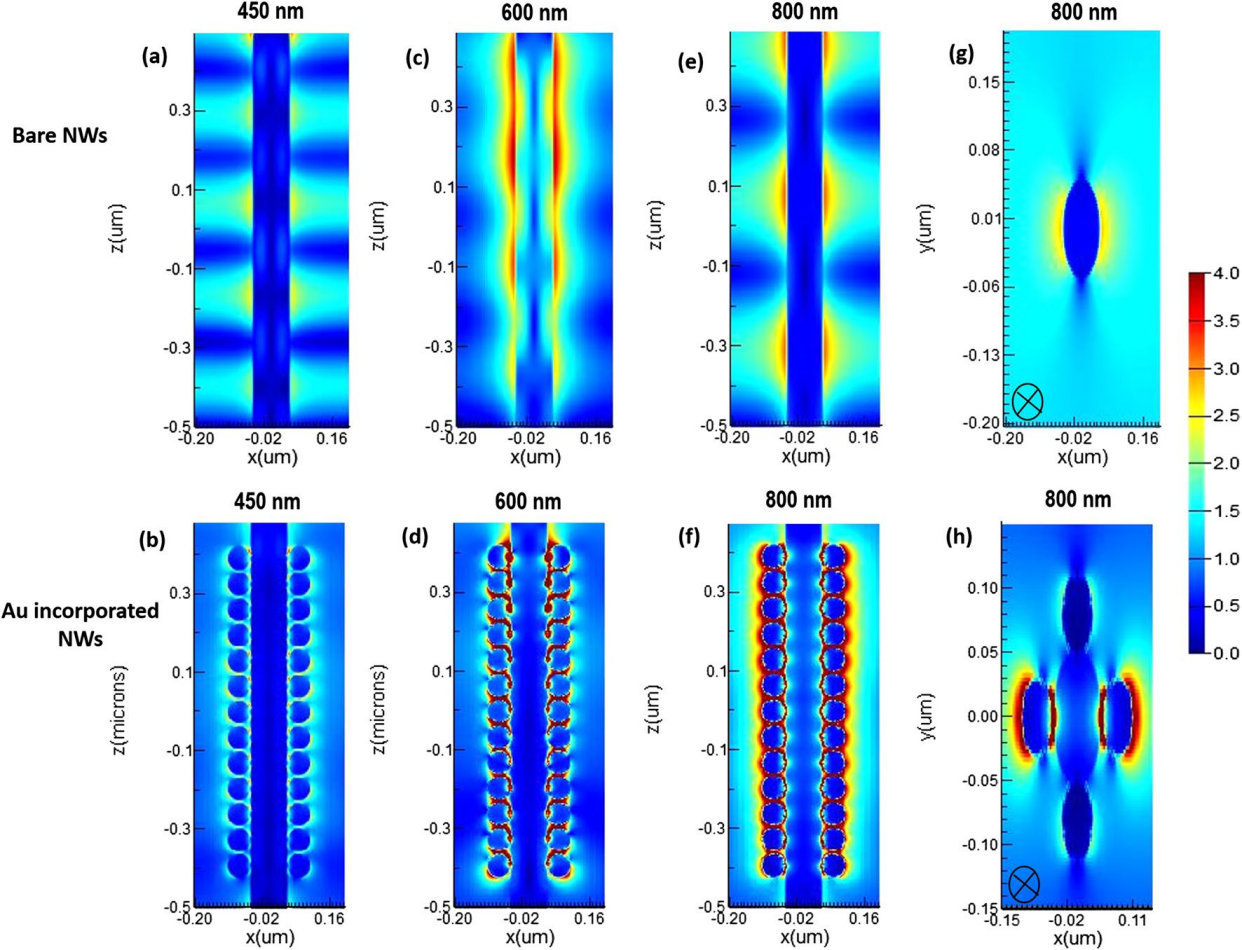
Side view of the 2D light distribution for the bare GaAs nanowire at wavelengths **a** 450, **c** 600, and **e** 800 nm compared to the GaAs NW decorated with 60 Au NPs (**b**), (**d**), and (**f**). Figures **g** and **h** show the top view from Figures (**e**) and (**f**), respectively

The optimal choice of NW geometry or the ratio of fill diameter-to-periodicity (*D*/*P* ratio) enables highly efficient absorption of the solar cell. Therefore, we optimized the *D*/*P* ratio of the NW by optical simulation to achieve the best optical absorption characteristics in a GaAs nanowire array solar cell with *p*-*i*-*n* junction. Figure 2 shows the total absorbed power of a bare GaAs nanowire with a length (*L* = 1 µm) and diameter (*D* = 100 nm), at different periodicity between 165 and 500 nm and aspect ratio between 0.6 and 0.2. From Fig. 2, it can be seen that for wavelengths of 300–600 nm, the absorption efficiency of the NWs is maintained above 90% in all simulations regardless the NW *D*/*P* ratio, which is much higher than that for the material thin films. For the NW *D*/*P* ratio of 0.2 (solid line), a sharp decrease in absorption occurs for photon energies smaller than the corresponding bandgap for bare GaAs NW. Above 600 nm to the wavelength close to the bandgap, Fig. 2 shows that the absorption of the NW is strongly influenced by increasing the *D*/*P* ratio. The best absorption spectrum was obtained at a *D*/*P* ratio of 0.6 (hollow circles). As the NW periodicity decreases with the increase of *D*/*P* ratio, Fig. 2 shows that the light trapping effect of the NWs decreases drastically at the wavelength close to the bandgap for lower NW *D*/*P* ratios. It has been demonstrated in the literature that the *D*/*P* ratio plays a significant role in the absorption of GaAs NWs [34, 35]. FDTD calculations reveal that the optical absorption of NWs is sensitive to geometrical parameters such as NW diameter, length and larger *D*/*P* ratio. However, in combination with metal NPs, the absorption of NWs with a lower *D*/*P* ratio at the wavelength near the bandgap improves more significantly than that with a higher NW *D*/*P* ratio. Motivated by this observation, we performed optical simulations for our GaAs NW structures incorporated with different NPs sizes at smaller *D*/*P* ratios of 0.2 and 0.3, respectively. As a typical example, Fig. 3 shows the calculated total absorption power for GaAs NWs at a *D*/*P* ratio of 0.2 incorporated with different Au- NP diameters of 30 nm (filled dots), 40 nm (filled squares), 50 nm (filled triangles), and 60 nm (hollow circles), respectively. For comparison, the absorption of the bare NW is also plotted (solid line). From Fig. 3, it can be deduced that when the Au NPs are introduced, a NP size-dependent field enhancement within the NW is well established. This is probably due to the resonant coupling of the free conduction electrons, referred to as plasmons, which leads to enhanced absorption within the NW. We found that as the size of the incorporated NPs increases, the NW absorption is effectively enhanced, most significantly at the light wavelength above the cutting edge from 650 nm to the near-bandgap wavelength of 800 nm. The best absorption within the NW is achieved when 60 nm Au NPs diameter is incorporated. On the other hand, at short wavelengths of 300–400 nm, the simulation shows a modest drop in absorption performance of almost 20–30% after the incorporation of the full-size range of Au NPs. In addition, a sharp decrease in absorption power occurs at wavelengths corresponding to the plasmon resonance of the incorporated Au NPs (wavelengths of 440–470 nm). This is likely due to localized surface plasmon resonances (LSPRs) confined within the NPs. Next, we investigated the field distribution inside the NW at the near-bandgap wavelength of 800 nm, where the optical absorption of the NW is effectively enhanced by the surface plasmons. We compared the light distribution inside the NW structure before and after decorating the NWs with the Au NPs, as shown in Fig. 4. The latter shows top view of the 2D intensity distribution in the *x*–*y* plane over the cross section of a GaAs NW obtained from the simulation top monitors for the electric field |*E*| (a), and the total absorbed power (b), at the wavelength of 800 nm before and after decoration with Au NPs with diameters of 30, 40, 50, and 60 nm, respectively. The color bar indicates the field intensity normalized to the maximum value. From the results, it can be seen that for small NP size, the trapped electric field belongs to the low-order localized surface plasmon mode, while as the diameter of NP increases, the higher-order mode is excited. From Fig. 4, it can be seen that the light coupling from the Au NPs in the *x*-direction into the neighboring GaAs NW is readily apparent and most obvious when the size of the incorporated Au NPs increases. In contrast, no effect of field enhancement and/or light coupling into the NW is found from the NPs in the *y*-direction. The collective oscillations of the NPs seem to be concentrated on the forward and backward directions of the NPs rather than on the coupling into the NW. When the *D*/*P* ratio of the NW filling is increased to 0.3 (Fig. 5) and Au NPs with diameters of 40 nm (filled circles), 50 nm (filled triangles), and 60 nm (hollow circles) are incorporated, respectively, the overall absorption efficiency of the NW remains above 95% for the different incorporated NP sizes. Compared to Fig. 3, a slight decrease in the absorption efficiency is observed for wavelengths corresponding to the plasmon resonance of the incorporated NPs in the range of 440–470 nm. As the size of the incorporated Au NPs increases, the NW absorption is effectively enhanced, and again most significantly between the wavelengths of 650 nm—up to the GaAs bandgap edge. Moreover, the best NW absorption is found when 60 nm Au NP diameter is incorporated. The simulation results in Figs. 3, 4 and 5 strongly suggest that the incorporation of the Au NPs within the NWs leads to greatly enhanced absorption of the GaAs NWs, even at small *D*/*P* ratios where the absorption of the bare NWs is lower as expected. The LSPR that occurred at the surface of the Au NPs is probably the main source of the enhanced local field within the aligned GaAs NWs. The LSPR is strongly dependent on the NP size, shape, and surrounding material properties [13]. To clarify the plasmon-enhanced NW absorption in more detail, we investigated the GaAs NW field enhancement when decorated with a single NP with a diameter of 60 nm, which found to have the best results. We set the NW periodicity to 0.2 and chose three typical light wavelengths of 450, 600, and 800 nm. At these light wavelengths, the NP decoration probably affects the NW absorption. We compared the light distribution within the NW structure before and after decoration with the NPs, as shown in Fig. 6a–h. Figure 6a shows a side view of the 2D electric field strength at a wavelength of 450 nm for bare GaAs NW calculated by FDTD. As can be seen, the light distribution of the bare NW in Fig. 6a shows a nice absorption profile at the top, middle, and bottom of the NW. On the other hand, the simulation of the Au-incorporated GaAs NW in Fig. 6b shows a little effect on the NW absorption, i.e., the incident light is hardly absorbed along the entire length of the NW. The weak *E*-field distribution within the NW indicates poor light absorption. In addition, the light field is rather concentrated around the Au NPs than within the NW. This is probably due to the lower extinction coefficient of the excited LSPR in the near field [15]. Figure 6c shows the light distribution for bare GaAs at 600 nm wavelength. The Figure illustrates that most of the incident light is absorbed in the upper half of the GaAs NW. After decoration with the Au NPs, Fig. 6d shows an improved absorption profile compared to Fig. 6b. A small fraction of the E-field is uniformly distributed with higher intensity along the entire length of the NW, with a tendency to concentrate at the top of the NW. Moreover, Fig. 6d shows that the excitation transfer is dominant within the NPs. At 800 nm wavelength, the absorption of the bare NW shows a uniform field distribution on the top, middle and bottom of the whole NW, as shown in Fig. 6e. On the other hand, the NW absorption is greatly enhanced after decoration with Au- NP, and the absorbed field intensity within the GaAs NW remains almost unchanged from the top to the bottom of the NW (Fig. 6f). Moreover, a concentrated field around the NPs can be easily seen. Figure 6g and h shows the top view of the 2D E-field distribution within the GaAs NW at 800 nm, as shown in Fig. 6e and f, respectively. Given that our study focuses only on decorating the GaAs NWs with Au NPs, compared to published literature results [34], our findings indicate that the metal NPs do improve the absorptance of GaAs NWs even at lower *D*/*P* ratio, i.e., of 0.2. The advancement of our results is the possibility to further enhance the absorption of the NWs at higher wavelengths, i.e., 600 and 800 nm.

**Fig. 7:**
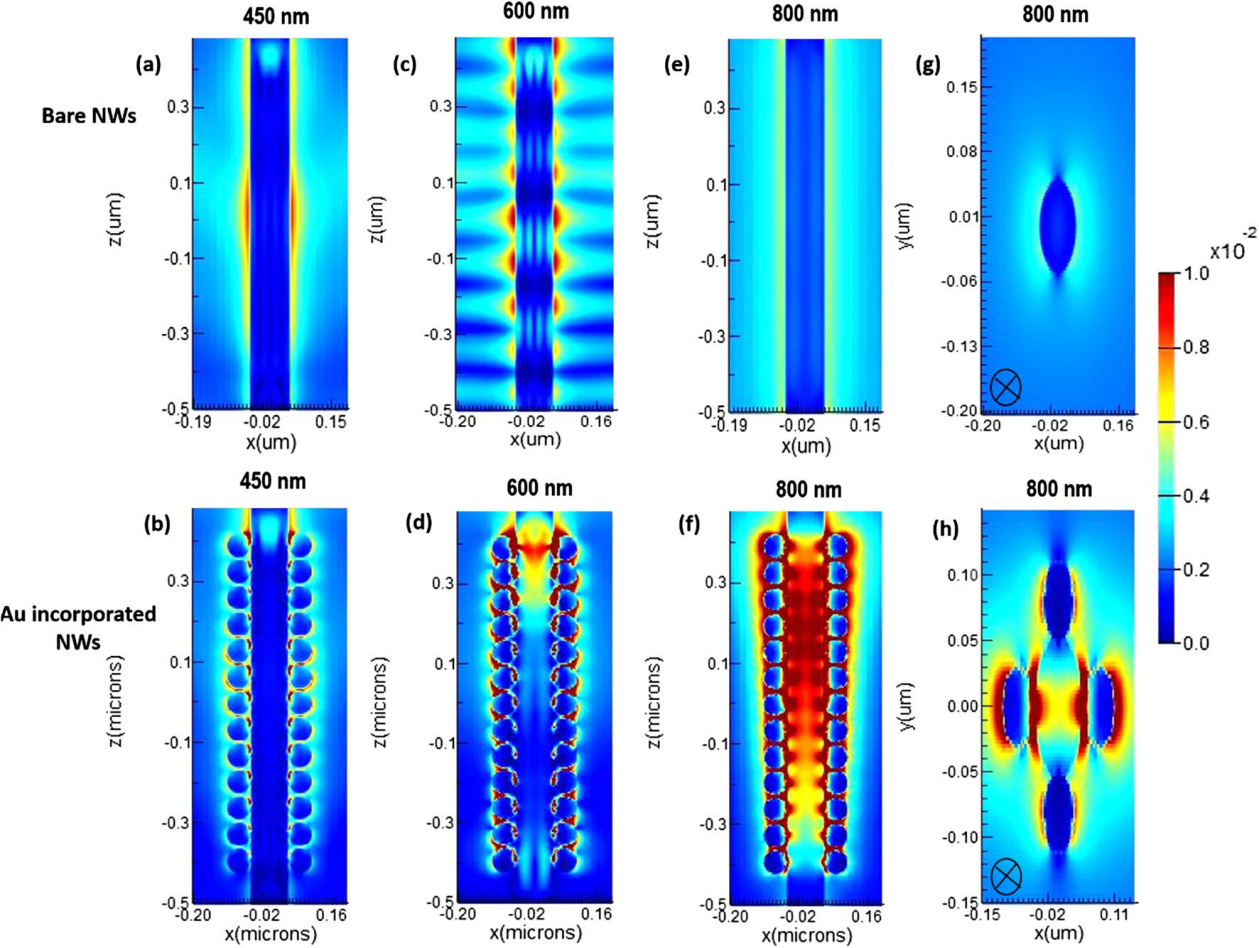
Side view of 2D absorption power distribution in bare GaAs at wavelengths **a** 450, **c** 600 and 800 nm **e** compared to NPS-decorated GaAs NW (**b**), (**d**) and (**f**). Images **g** and **h** show the top view from images (**e**) and (**f**), respectively

**Fig. 8:**
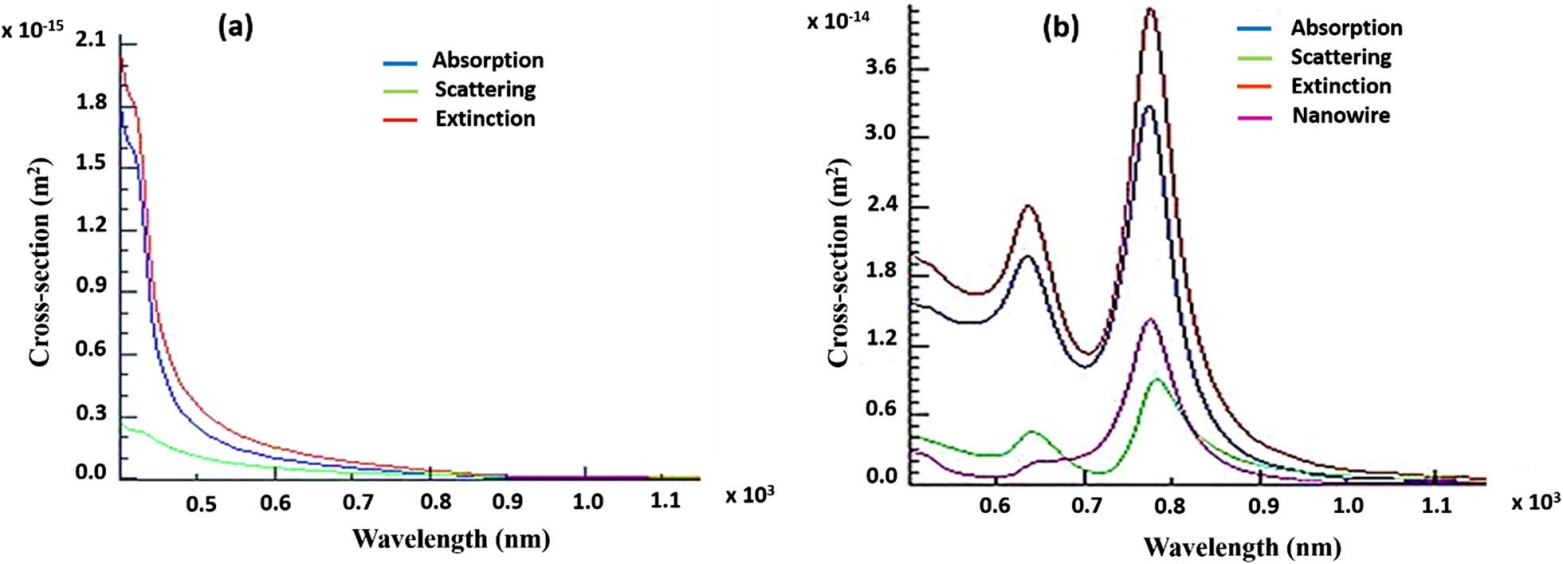
The absorption, scattering, and extinction cross sections (absorption + scattering) for bare GaAs NW **a** under perpendicular illumination and **b** for NW decorated with 60 Au NPs (maximum 26 NPs), respectively

**Fig. 9:**
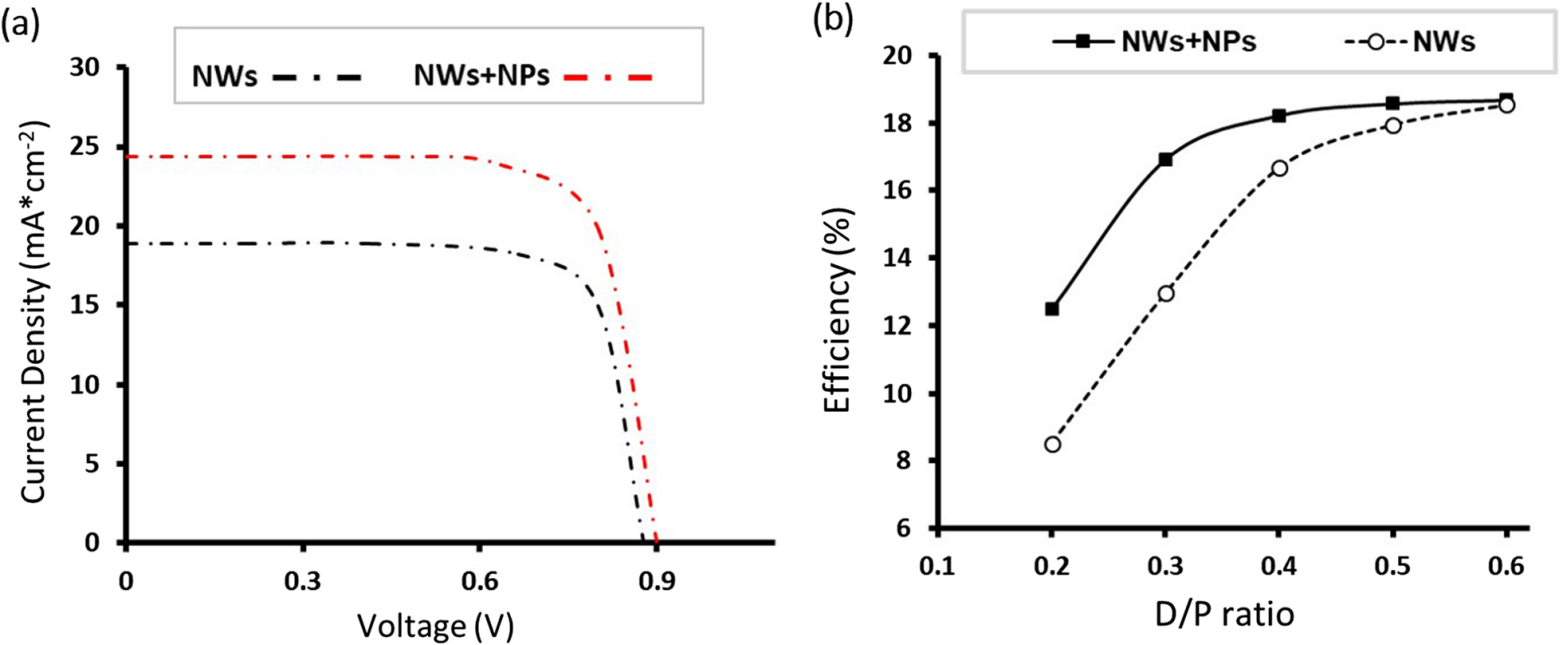
**a** Comparison of *I*–*V* characteristics between bare NWs and NWs with 60-nm Au NPs; **b** photoconversion efficiency of NWs with different *D*/*P* decorated with 60-nm Au nanoparticles

To complete the analysis set, the total absorbed power within the GaAs NW is calculated after decorating the 60 nm Au NPs diameter at the three cutting light wavelengths of 450, 600 and 800 nm (Fig. 7a–f). Again, the total absorbed power for the bare NW at these three light wavelengths is included for comparison. From Fig. 7a–f, it can be concluded that increased absorbed power is seen within the NW after decoration of the Au NPs, especially at the higher wavelengths of 600 and 800 nm, compared to the bare GaAs NW. The best absorbed power is found for the GaAs-Au decorated NW at 800 nm wavelength (Fig. 7f). For the latter, the absorbed power distribution is strongly increased in the upper half of the GaAs NW, which is consistent with the previous results in Fig. 3. Figure 7g, h shows the top view of the 2D *E*-field distribution within the GaAs NW at 800 nm, as shown in Fig. 7e, f, respectively. These simulation findings indicate that the light concentration due to the excitation of LSPR around Au NPs leads to enhanced localized photocurrents in the GaAs NW, enabling them to serve as effective nanoscale energy transfer antennas for the incident light. To gain further insight into the absorption efficiency of the nanowires, the extinction cross-sectional data (absorption + scattering) of the GaAs NWs before and after decoration with the 60 nm Au NPs were calculated. Figure 8a, b shows the optical extinction cross section for bare GaAs nanowires (a) and Au decorated nanowires (b) under perpendicular illumination. Figure 8a indicates a maximum absorption of the bare NW at a wavelength of about 400 nm. The latter explains quite well that the GaAs NWs are good absorbers in the UV region of the EM spectrum. Moreover, the extinction coefficient in Fig. 8a is dominated by NW absorption, while light scattering is minimal. Figure 8b shows the simulated optical extinction cross section of GaAs NW decorated with Au NPs of 60 nm diameter. As can be seen, the light-trapping ability of GaAs NW shows two absorption peaks as follows: (1) In the near-infrared region; the NW absorption occupies ~ 8% at 650 nm wavelength. These are presumably the LSPRs confined on the sidewall around the NW; (2) In the far field, the NW absorption occupies ~ 35% at ~ 800 nm wavelength while maintaining a higher optical extinction coefficient. Comparing Fig. 8a, b, it can be deduced that the optical cross section is effectively increased. An order of magnitude increase in the optical cross section is readily apparent. Next, the optical generation and photoconversion efficiency of our solar cell structure before and after decoration with NPs are investigated. Figure 9a shows the efficiency of the NW solar cell structure with (red line) and without decoration of Au NPs (black line) under AM 1.5G illuminations. We clearly observe an increased photocurrent as a result of decoration with Au NPs. The open circuit voltage (*V*_oc_) increases slightly from 0.878 (for bare NWs) to 0.899 (for decorated NWs). Moreover, the short-circuit current density (*J*_sc_) increases dramatically from 18.9 (for bare NWs) to 24.3 mA/cm^2^ (for decorated NWs). Figure 9b shows an increasing photoconversion efficiency with increasing *D*/*P* ratio (maximum at 0.6). The Figure shows that the photoconversion efficiency increases with increasing *D*/*P* ratio up to values between 0.5 and 0.6, above which stability of photoconversion efficiency is achieved. This is presumably due to the fact that the incident light in a full wavelength band can be absorbed by GaAs nanowires if the *D*/*P* ratio is large enough. Moreover, the reflection increases at high *D*/*P* ratios, which would decrease the absorption efficiency. From the Figure, it can be seen that the efficiency of the new structure is improved by a factor of 24 from 12.96 to 16.92% when the *D*/*P* ratio is 0.4. As the photoconversion efficiency seemingly influenced by many factors, it is conceivable from our results that the improvement in photocurrent density is due to the incorporation of Au NPs within our NWs structures. The latter provides a method to improve the light trapping at lower *D*/*P* ratios of the GaAs NWs material. Our study combining LSPRs with nanowire arrays, both of which have obvious effects on light trapping, provides insight into further research to improve solar efficiency and may reduce the cost of solar cells if further optimized.

Following light absorption, we put forward three possible mechanisms of plasmonic enhancement within the NW, employing (1) scattering of incident photons, (2) charge carrier transfer, and (3) near-field enhancement. Considering mechanism (1), our NPs with 60 nm diameter have a sufficiently large volume to scatter light efficiently. This is because the intensity of the scattered light varies with the sixth power of the particle diameter [39]. In this respect, the plasmonic NPs act as nanoreflectors for incident photons in the forward and backward directions through absorption and reemission mechanisms [40]. The latter leads to a lengthening of the mean photon path, resulting in an increasing trapping rate of incident photons. As for mechanism (2), in the plasmonic semiconductor-NW combination, a Schottky barrier localized at the interface blocks the transfer of electrons from the NP to the NW and vice versa. However, if the absorbed energy of hot electrons upon LSPR excitation of the metal NPs is sufficient, the electrons can overcome the barrier and be injected into the conduction band of the NW. In this picture, mechanism (2) contributes to the plasmonic enhancement of light absorption within the vertically aligned GaAs nanowire. In addition, incident light is absorbed in a suitable spectral range with simultaneous overlap of LSPR and bandgap energy can substantially trigger the bandgap excitation of the semiconductor. From this point of view, enhanced rates of electron–hole generation can be achieved in the NW semiconductor exposed to the electric field in mechanism (3). Moreover, the immobilization of Au NPs in contact with the semiconductor NW can often facilitate the charge separation in electron–hole pair generation, since the Fermi levels of the plasmonic NPs are much lower than those of the conduction band edges of the semiconductors [41]. Since the mechanism of hot-carrier injection requires that the metallic NPs and the NW are in ultimate contact, it has been found that the carrier generation of the semiconductor is enhanced by the LSPR of the metal even under electrical isolation [42–50]. A strong electric field is observed in the vicinity of the NPs; the intensity of which is several orders of magnitude larger than that of the incident far field [41]. The latter has been vividly demonstrated in an optical simulation study using the finite-difference time-domain (FDTD) method [51].

## Conclusions

In summary, a novel plasmon-enhanced solar cell structure based on a GaAs nanowire array decorated with Au nanoparticles is presented. The results of the GaAs NW absorption are evaluated for NW diameter (*D* = 100 nm), (*L* = 1 μm), and (*D*/*P* = 0.2–0.6). Our calculation shows that the best absorbed power for the GaAs NW occupies ~ 35% at ~ 800 nm wavelength when decorated with 60 nm Au nanoparticles, which is much higher than that of thin films. Moreover, the simulated optical generation in the GaAs nanowire is concentrated in the upper half of the nanowire, dominated by the excitation transfer. The LSPR occurring at the surface of the Au nanoparticles is believed to be the main source of the enhanced local field within the aligned GaAs nanowires. The concentrated incident light leads to an increase in the electron–hole pair generation rate within the nanowires, thus improving the overall efficiency of the solar cell. The structure explains quite well that the GaAs nanowires are good absorbers in the UV region of the EM spectrum. Our study combining LSPR with nanowire arrays provides an exciting tool for further research to reduce the cost of solar cells.
